# Breast cancer detection based on histological images using fusion of diffusion model outputs

**DOI:** 10.1038/s41598-025-05744-0

**Published:** 2025-07-01

**Authors:** Younes Akbari, Faseela Abdullakutty, Somaya Al Maadeed, Ahmed Bouridane, Rifat Hamoudi

**Affiliations:** 1 Department of Computer Science and Engineering, Qatar University, Doha, Qatar; 2 Center for Data Analytics and Cybernetics, University of Sharjah, Sharjah, UAE; 3 BIMAI-Lab, Biomedically Informed Artificial Intelligence Laboratory, University of Sharjah, Sharjah, UAE; 4 Research Institute for Medical and Health Sciences, University of Sharjah, Sharjah, UAE; 5 Clinical Sciences Department, College of Medicine, University of Sharjah, Sharjah, UAE

**Keywords:** Breast cancer, Biomedical engineering

## Abstract

The precise detection of breast cancer in histopathological images remains a critical challenge in computational pathology, where accurate tissue segmentation significantly enhances diagnostic accuracy. This study introduces a novel approach leveraging a Conditional Denoising Diffusion Probabilistic Model (DDPM) to improve breast cancer detection through advanced segmentation and feature fusion. The method employs a conditional channel within the DDPM framework, first trained on a breast cancer histopathology dataset and extended to additional datasets to achieve regional-level segmentation of tumor areas and other tissue regions. These segmented regions, combined with predicted noise from the diffusion model and original images, are processed through an EfficientNet-B0 network to extract enhanced features. A transformer decoder then fuses these features to generate final detection results. Extensive experiments optimizing the network architecture and fusion strategies were conducted, and the proposed method was evaluated across four distinct datasets, achieving a peak accuracy of 92.86% on the BRACS dataset, 100% on the BreCaHAD dataset, 96.66% the ICIAR2018 dataset. This approach represents a significant advancement in computational pathology, offering a robust tool for breast cancer detection with potential applications in broader medical imaging contexts.

## Introduction

Breast cancer, with approximately 2.3 million new cases annually, stands as the most prevalent cancer type worldwide. Advances in molecular medicine have identified breast cancer as a heterogeneous disease comprising distinct subtypes, including triple positive, triple negative, luminal A, and luminal B, which are characterized by biomarkers such as HER2, ER, and PR^1,2^. Each subtype exhibits unique histopathological features that contribute to variations in disease progression and treatment response. Whole slide images (WSIs) have become indispensable in evaluating critical disease aspects such as staging, metastasis, and prognosis. The advent of digital slide scanners has transformed histopathology by enabling rapid digitization of slides, thereby streamlining storage, sharing, and computational analysis^3–5^. However, the WSIs have considerable problems that make them difficult to analyse. The varying resolution of different scanners from 0.25 µ/pixel to coarser scales can affect the visibility of fine histological details such as nuclear morphology, which can lead to inconsistencies in segmentation and recognition results. In addition, biases in the dataset, such as overrepresentation of certain tissue types or staining variations due to differences in dissection, can distort the computational models and reduce their generalizability to different patient populations. Accurate detection and segmentation of tissue regions in WSIs are vital for improving breast cancer detection and understanding its relationship with patient prognosis. Aggressive breast cancer subtypes, for instance, often display characteristic histological features, such as vesicular nuclei and prominent nucleoli^6^. Developing a robust quantitative pipeline for nuclear analysis is essential for exploring the tumor microenvironment (TME) and gaining deeper insights into tumor behavior and patient outcomes^7,8^.

**Fig. 1:**
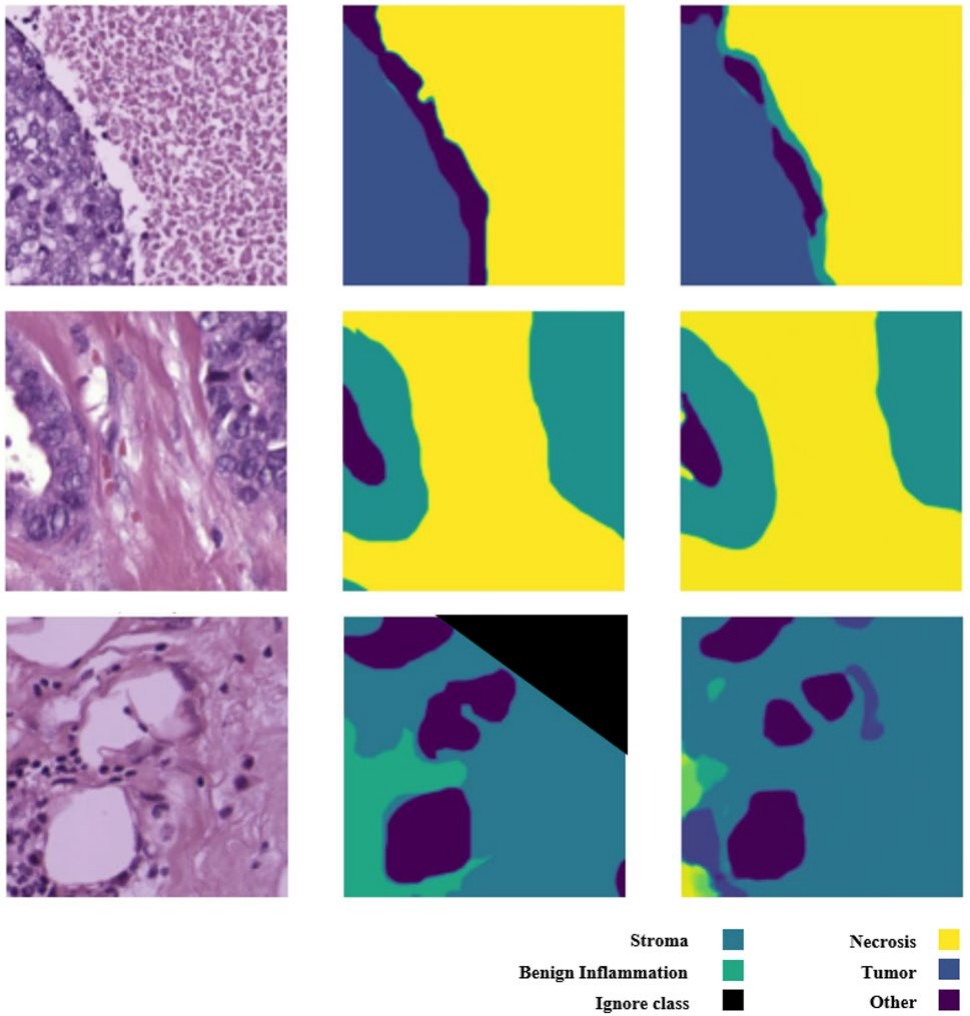
Breast cancer tissue regions segmentation. (first column) original pathological images, (second column) ground truth based on tissue regions such as Tumor, Stromal, Necrosis and Other. (third column) predicted region by DDPM.

Accurate delineation of tissue regions is critical for advancing computer-aided diagnosis, prognosis, treatment response evaluation, and understanding cancer biology^9,10^. However, this task is inherently difficult due to the diverse spatial distributions, irregular morphological variations, and indistinct boundaries between different tissue types. The tumor microenvironment (TME) adds another layer of complexity, being a heterogeneous ecosystem comprising tumor epithelial cells, cancer cells, fibroblasts, inflammatory cells, tumor-infiltrating lymphocytes (TILs), and tumor-associated stroma. Precise identification and segmentation of these components are pivotal for accurate TME quantification and for enhancing the reliability of breast cancer detection.

Relying exclusively on predicted cancer tissue for breast cancer detection may introduce significant errors due to the inherent intricacies of the TME and tissue interactions. The segmentation challenges are exemplified in Fig. 1, which highlights the complexity of these tissue distributions. Semantic segmentation aims to classify each pixel within an image into specific tissue categories, distinguishing it from nuclei segmentation, which focuses on identifying individual nuclei. Tissue semantic segmentation extends beyond this to classify regions based on their tissue types, encompassing multiple nuclei and the surrounding stroma^11,12^.

**Fig. 2:**
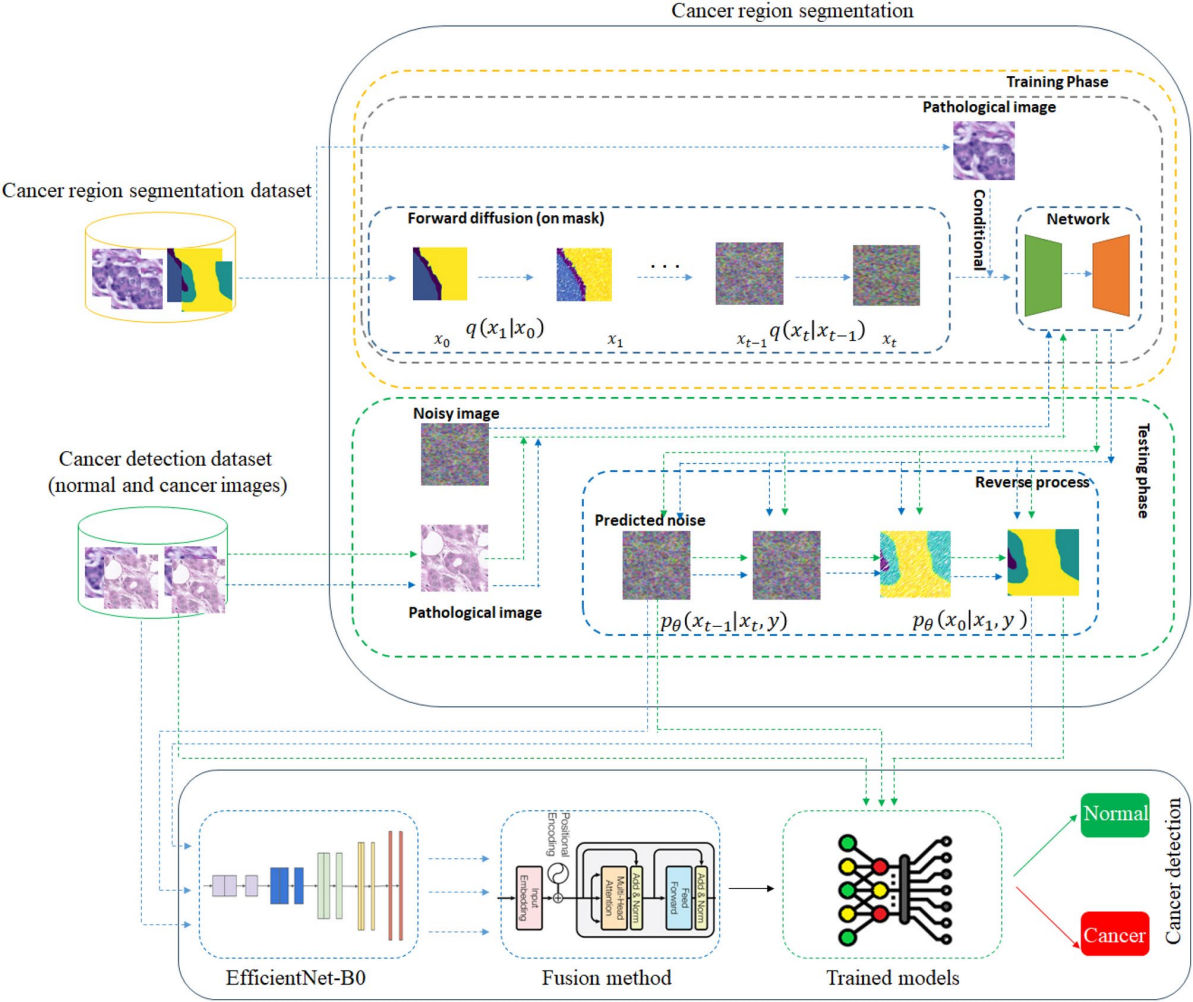
Architecture of the proposed method: the denoising diffusion probabilistic model (DDPM) for pathological image segmentation and EfficientNet-B0 and transformer decoder as Breast Cancer Detection.

In the figure, while the first two rows demonstrate accurate segmentation of tissue regions, the third row reveals an instance where tumors are misclassified, highlighting the potential for false detections. Specifically, certain regions are mistakenly labeled as “Stroma” or “Other” instead of tumor tissue. These misclassifications stem from multiple challenges, including boundary ambiguity between tumor and adjacent stroma tissues and staining variability across Whole Slide Images (WSIs). These errors highlight a critical limitation in relying solely on segmentation results, which may compromise the reliability of the detection system and lead to missed diagnoses or false positives in clinical settings. To address these challenges, we introduce a comprehensive approach that combines three complementary sources of information: predicted segmentation regions, original image data, and the noise inferred during segmentation. Our approach leverages a Conditional Denoising Diffusion Probabilistic Model (DDPM) for tissue segmentation, which enables us to capture supplementary information through the noise predicted during the denoising process. This noise contains valuable signal about areas of uncertainty in the model’s predictions, potentially highlighting regions that merit closer examination. By integrating all three information sources, as illustrated in Fig. 2, our system achieves more robust tumor detection that is less susceptible to the segmentation errors.

For segmenting tissue regions, we utilize one of the most advanced methods available, namely DDPM. Diffusion models, including DDPM^13,14^, have demonstrated remarkable success across various domains such as segmentation^15,16^, super-resolution^17^, object detection^18^, and crowd counting^19^. Building upon the strengths of diffusion models, we propose an innovative framework designed to enhance classification performance, particularly for pathological images. Our method integrates a conditional diffusion model for region segmentation with a CNN-based approach for breast cancer detection. The Breast Cancer Semantic Segmentation (BCSS) dataset^9^, containing tissue-based histopathology images, is employed to evaluate our approach. The diffusion process begins by using the mask of the pathological image as input, with gradual noise addition. The pathological image is subsequently conditioned with the noisy image, serving as input to the denoising network for noise prediction. In the classification phase, noisy images and pathological images from breast cancer detection datasets are conditioned through the trained denoising network to derive the predicted noise. This noise undergoes the reverse diffusion process to generate the predicted segmentation mask. Finally, the original image, predicted region, and noise are fed into the EfficientNet-B0 model. The extracted features from these inputs are then processed through a transformer decoder, fusing the three feature types to classify images as either normal or cancerous. To validate the efficacy of our method, we provide empirical analyses demonstrating that this integrated approach significantly enhances breast cancer detection in pathological images.

In summary, our contributions can be outlined as follows:

- An empirical demonstration of the effectiveness of the approach for image classification, with a particular emphasis on pathological images.
- A novel fusion approach using DDPM enhances breast cancer detection in histopathology.
- Extensive experiments using four public datasets, resulting in a superior method of classification, surpassing existing state-of-the-art techniques.

## Related works

This section examines both tissue region segmentation and cancer detection based on pathological images of breast cancer.

### Tissue region segmentation

The main goal of histopathological image analysis in breast cancer research is to improve diagnostic and prognostic accuracy through advanced segmentation methods. While deep learning has shown promise in this area, it continues to encounter significant challenges. This discussion provides a detailed overview of the growing literature on histopathologic image analysis in breast cancer.

Segmentation tasks, such as region identification, play a central role in histopathologic image analysis of breast cancer. A widely used resource in this context is the Breast Cancer Semantic Segmentation (BCSS) dataset^9^. This review focuses on the latest techniques that have been developed and applied to this dataset and highlights advances in the field.

Lopez et al.^20^ utilized the BCSS dataset^9^ to create a novel crowdsourcing (CR) segmentation approach that combines a segmentation network (SN) with an annotator network (AN). Their method was compared with established techniques such as STAPLE^21^. Its strength lies in its ability to handle noisy annotations, making it adaptable to real-world datasets. However, it struggles with computational complexity due to the dual-network architecture and may not generalize well to datasets with significantly different tissue distributions. He et al.^11^ proposed DETisSeg, a dual-encoder network that merges global context from a Swin transformer branch with local features from a CNN branch. DETisSeg utilized enhanced residual connections and a pyramid architecture decoder, showing superior performance compared to state-of-the-art methods like SwinUnet^22^ and the Swin Transformer^23^. Its strength is its multi-scale feature extraction, but it requires substantial computational resources and may falter with class-imbalanced datasets, such as those with underrepresented necrosis regions.

### Breast cancer detection

This subsection reviews recent advances in the detection of breast cancer using various machine learning and deep learning methods. Some studies have focused on direct detection of breast cancer using deep learning models^24–26^, while others have utilized ensemble learning techniques^27–30^. In addition, some research has included the segmentation of tumor heat maps as a precursor to detection^31–34^.

Sharma et al.^24^ demonstrated the utility of transfer learning by using the pre-trained Xception model for magnification-based classification of breast cancer histopathological images. This approach, paired with an SVM classifier using a radial basis function kernel, achieved classification accuracies of 96.25%, 96.25%, 95.74%, and 94.11% at magnification levels of 40×, 100×, 200×, and 400×, respectively. This approach excels in leveraging pre-trained features for efficiency but is limited by its dependence on magnification-specific training, reducing flexibility across varied imaging conditions. Rashmi et al.^25^ developed BCHisto-Net, a CNN model for classifying breast histopathology images at 100x magnification by extracting both global and local features. Their model achieved a classification accuracy of 89 on the BreakHis dataset^35^. While computationally lightweight, its performance drops with datasets exhibiting high heterogeneity, as it lacks advanced feature fusion.

Majumdar et al.^27^ proposed an innovative rank-based ensemble method that integrates predictions from GoogleNet, VGG11, and MobileNetV3_Small models using the gamma function, effectively addressing a binary classification problem. However, its complexity increases training time, and it may overfit small datasets. Similarly, Bhowal et al.^29^ introduced a fuzzy ensemble approach for classifier fusion that utilizes the Choquet integral method and employs coalition game theory and information theory to compute fuzzy measures. This method handles uncertainty well but requires careful tuning of fuzzy measures, limiting scalability Wang et al.^30^ presented a transfer learning framework that utilizes manifold learning and adaptive preservation techniques to fuse features at different CNN model levels to ensure robust performance. Its strength is adaptability, though it struggles with computational overhead and lacks explicit segmentation integration.

In the field of tumor heatmap segmentation, Zhang et al.^31^ proposed a three-stage pipeline with Cycle-GAN normalization, improved DPN, and Swin Transformer classification, achieving strong localization. However, its multi-stage design increases processing time, and it relies heavily on heatmap accuracy. Guo et al.^33^ proposed v3_DCNN, which integrates the Otsu algorithm for tissue filtering, Inception-v3 for preliminary tumor region selection, and DCNN for refined segmentation and localization. This method achieved an FROC value of 83.5% on the Camelyon16 dataset, offering efficiency but limited precision in complex tumor boundaries. Lin et al.^32^ ScanNet, a semantic segmentation model based on Fully Convolutional Networks (FCNs) that uses larger patches in testing to increase efficiency. Their method achieved an FROC value of 85.33% on the Camelyon16 dataset. Its speed is a strength, but it sacrifices detail in smaller regions.

These studies illustrate the different methods used in the detection of breast cancer and show the potential of both direct classification and segmentation-based approaches. Our proposed DDPM-based fusion method advances beyond existing approaches by integrating multi-modal data (original images, predicted segmentation masks, and diffusion noise) to capture complementary features, unlike single-modality methods like Xception^24^ or BCHisto-Net^25^, which rely solely on image features. Compared to segmentation-focused methods like DETisSeg^11^, our approach leverages DDPM’s noise predictions to enhance segmentation robustness in ambiguous regions, while the transformer decoder’s attention mechanisms outperform simpler fusion techniques^27,29^ by dynamically weighing multi-modal contributions, achieving higher accuracy and generalizability across diverse datasets.

## The method

The components of the segmentation and detection phases are elaborated upon in the following sections. In the segmentation phase, the BCSS dataset^9^ is utilized to obtain tissue regions. For the detection phase, the method is evaluated on four public datasets: ICIAR-2018^36^, BreCaHAD^37^, Invasive Ductal Carcinoma (IDC)^38^, and BRACS^39^. Detailed descriptions of these datasets are provided in section “Datasets”.

Our proposed approach integrates a Conditional Denoising Diffusion Probabilistic Model (DDPM) for tissue region segmentation with an EfficientNet-B0 network for feature extraction and a transformer decoder for feature fusion, synergistically improving breast cancer classification accuracy. Note that joint training is not applicable in our setup, as the segmentation model is trained on a separate dataset with pixel-level labels, while the classification datasets lack segmentation annotations. Hence, the segmentation module is fixed during classification training. The DDPM, detailed in section “Segmentation phase”, generates precise segmentation masks by denoising histopathological images conditioned on their original patterns, capturing critical tissue regions (e.g., tumor, stroma) that provide structural context often missed by direct classification methods. These masks, along with predicted noise and original images, form a rich, multi-modal input set. EfficientNet-B0, described in section “Detection phase”, extracts enhanced spatial and textural features from this trio of inputs, leveraging its efficient architecture to distill discriminative patterns across modalities. The transformer decoder then fuses these features, using self-attention and cross-attention mechanisms to weigh and integrate complementary information such as tumor boundaries from masks, subtle anomalies from noise, and holistic details from original images resulting in a robust representation for classification.

### Segmentation phase

This phase, which comprises four components: the forward process, the denoising network and the training and testing processes, is carried out using the BCSS dataset, a dataset for segmenting tissue regions in pathological breast cancer images. The output of the phase provides the predicted noise and the obtained mask of the regions. Here, $x_{0}$ represents the mask of the pathological image *y*, which is gradually transformed into a noisy pattern $x_{t}$ for $t \in \left\{ 1,2,3,\ldots , T \right\}$ by gradually applying Gaussian noise according to a predefined noise variance schedule $\beta \in \left\{ \beta _{1},\ldots , \beta _{T} \right\}$. Let us define $x_{noisy}$ as the predicted noise that serves as the output of the denoising network. The fraction of real noise $x_{real}$ in $x_{t}$ is predicted at each time step, conditional on the pathological image. The following sections explain the details of these components in the training phase.

#### Forward process

Diffusion models, which are inspired by principles of non-equilibrium thermodynamics^40^, are a class of likelihood-based models that operate through a Markov chain framework involving both forward and reverse processes. During the forward process, noise is gradually introduced to the data, while the reverse process removes this noise. The forward process is mathematically defined as follows:

1 $$\begin{aligned} q\left( \textrm{x}{\textrm{t}} \mid \textrm{x}{\textrm{t}-1}\right) =\mathcal {N}\left( \textrm{x}{\textrm{t}} \mid \sqrt{1-\beta _t} \textrm{x}{\textrm{t}-1}, \beta _t \textrm{I}\right) , \end{aligned}$$

where $\mathcal {N}$ denotes a Gaussian distribution and *I* represents the identity matrix. However, $x_{t}$ can also be derived from $x_{0}$ and a noise vector $x_{real} \sim \mathcal {N}(0, \textrm{I})$. In a Gaussian distribution, the mean ($\mu$) and variance ($\sigma ^2 \ge 0$) characterize the noise at each time step *t*. Specifically, at each step, a new image is generated from a conditional Gaussian distribution where the mean is $\mu _{t}=\sqrt{1-\beta _t} \textbf{x}_{\mathbf {t-1}}$ and the variance is $\sigma ^2 = \beta _t$. This is achieved by setting:

2 $$\begin{aligned} \textbf{x}{\textbf{t}}=\sqrt{1-\beta _t} \textbf{x}{\mathbf {t-1}}+\sqrt{\beta _t} x_{real}. \end{aligned}$$

By using the known formula for $t=\left\{ 1,\ldots , T \right\}$, we derive a computable expression:

3 $$\begin{aligned} q\left( x_t \mid x_0\right) =\mathcal {N}\left( x_t, \sqrt{\bar{\alpha }_t} x_0,\left( 1-\bar{\alpha }_t\right) I\right) =\sqrt{\bar{\alpha }_t} x_0+\sqrt{1-\bar{\alpha }t} x{real}. \end{aligned}$$

This equation allows for the calculation of noise at any step *t* (since $\bar{\alpha }_t$ is known from $\bar{\beta }_t$), bypassing the need to follow the entire forward process sequentially.

#### Denoising network

The denoising network takes in a noisy image at any given time step and outputs a predicted noise that corresponds to the noise applied to $x_{0}$, following the pre-defined noise schedule $\beta _t$. The architecture of this denoising network is based on U-Net^13^, where the features are scaled down during downsampling layers and upscaled during upsampling layers, reducing the spatial dimensions by a factor of two at each step. At every stage, the network integrates an embedding sub-process that injects time-specific information. This is achieved using sinusoidal position encoding to represent the current time step *t*. The core building block of this network is a simple and linear ResNet block, which is embedded into both the downsampling and upsampling paths. Notably, the first downsampling block can not only accept input from the previous layer but also incorporate time step-related information. Additionally, in this implementation, certain ResNet blocks from the original U-Net architecture have been replaced by Attention blocks to enhance the model’s performance. Once the upsampling process is complete, the network produces the predicted noise $x_{noisy}$.

#### Training process

To train the proposed model, the denoising network is tasked with learning to map the input pathological image to the corresponding noise. This is done by minimizing the Euclidean distance between the actual noise ($x_{real}$) and the predicted noise ($x_{noisy}$). The loss function used for this purpose is the Mean Square Error (MSE), which quantifies the difference between the real and generated noise. It is defined as follows:

4 $$\begin{aligned} L_{\textrm{diff}}=\frac{1}{m} \sum _{i=1}^m \left\| x_{real}-x_{noisy}\right\| _2^2 \end{aligned}$$

Here, *m* represents the number of samples in the batch, and the loss function ensures that the model accurately predicts the noise applied at each time step. The training process is significantly influenced by the noise schedule and hyperparameters, which dictate the quality of segmentation performance. The noise schedule, defined by the variance terms $\beta _t \in \{\beta _1, \ldots , \beta _T\}$, controls the rate at which noise is added during the forward process and subsequently removed during denoising. In this study, we adopt a linear noise schedule over $T = 1000$ diffusion steps (see Table 1), as recommended by Ho et al.^13^, to balance gradual noise corruption with sufficient detail preservation. A linear schedule ensures a smooth transition from the original mask ($x_0$) to pure noise ($x_T$), allowing the model to learn robust noise predictions across all time steps. Alternative schedules, such as cosine-based schedules^14^, could prioritize early-step detail retention but may compromise performance on highly noisy inputs; our experiments confirmed that the linear schedule optimizes segmentation accuracy (e.g., IoU and Dice metrics) for the BCSS dataset’s complex tissue structures. Key hyperparameters further shape the training dynamics. The learning rate, set to $1 \times 10^{-4}$ with the AdamW optimizer, ensures stable convergence by balancing gradient updates, avoiding overshooting in the high-dimensional loss landscape typical of diffusion models. A warmup period of 5000 steps (Table 1) gradually increases the learning rate, enhancing early-stage stability when the model learns coarse noise patterns. The batch size of 16 strikes a compromise between computational efficiency and gradient noise reduction, enabling effective generalization across the diverse histopathological images in the BCSS dataset. The detailed training process is outlined in Algorithm 1.

**Algorithm 1:**
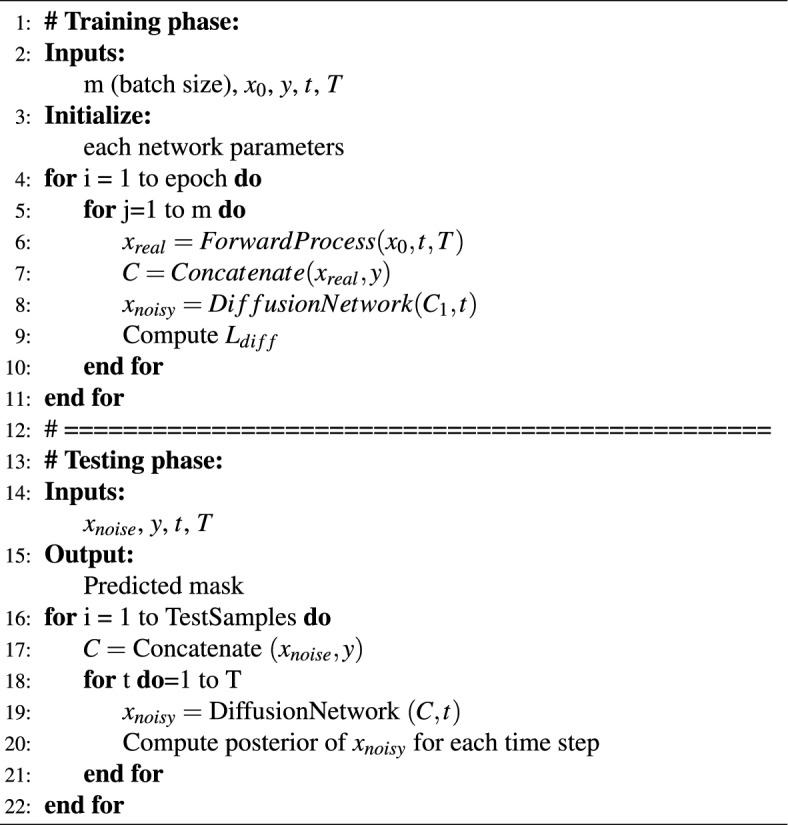
Training and testing phases

#### Testing process

In this step, four breast cancer detection datasets (including both training and testing data) are used to predict noise and segment regions. Unlike the training phase, the forward process cannot be applied to the test data since the mask information should not be available. Instead, the reverse process is employed, where the model predicts noise and refines the image. The diffusion model forecasts the total noise that must be removed at each time step, with a portion subtracted during each iteration of the noise prediction by the neural network. For example, at each time step, a noisy image ($x_{noise}$) combined with a histopathology image undergoes reverse processing. This reverse diffusion process calculates the posterior distribution of the noisy mask, progressively converting the noisy image back into its clean state. The objective is to transform pure noise into a segmented image, and this requires estimating the distribution of possible images. To approximate this conditional probability distribution, the trained denoising network is used, represented as $p_{\theta }(x_{t-1} \mid x_{t},y)$, where $\theta$ are the network parameters updated using gradient descent. Assuming the reverse process follows a Gaussian distribution, each Gaussian requires parameters for both its mean and variance. The full testing procedure is detailed in Algorithm 1.

### Detection phase

In the phase, two main steps are involved: EfficientNet-B0^41^ and transformer decoder^42^ to fuse the three types of data (original, mask, and predicted noise).

EfficientNet-B0 is a convolutional network architecture that belongs to the EfficientNet family. EfficientNet models are known for their ability to achieve high accuracy while being computationally efficient. They use a method called compound scaling, which scales the depth, width, and resolution of the network in a balanced way. They use mobile inverted bottleneck convolution (MBConv) blocks^43^, which are efficient both in terms of memory and computation. The model uses the swish activation function^44^, which increases the non linearity and improves the performance of the model. EfficientNet’s efficacy in breast cancer detection is well-established in the literature. Notably, Guo et al.^45^ demonstrated its capabilities by enhancing the architecture specifically for lymph node metastasis detection, achieving significant improvements in sensitivity and specificity. Further validating its versatility, Smith et al.^46^ developed the EarlyNet framework, which strategically pairs EfficientNet with VGG11 to optimize early-stage breast cancer classification, resulting in superior diagnostic accuracy compared to single-model approaches. In this work, we utilise the EfficientNet-B0 architecture with pre-trained weights to improve the accuracy and efficiency of our breast cancer detection model. To extract features for the three data types, we use the model that is the best model in our experiments.

Then, the features are concatenated and fed into a fusion method. The best fusion method in our experiments is the Transformer Decoder. The Transformer Decoder layer, a core component of the Transformer architecture presented by Vaswani et al.^42^, is used in our framework for feature fusion. This layer plays a crucial role in integrating features extracted from different types of data, thus improving the accuracy of breast cancer detection in histologic images. The Transformer Decoder layer consists of several key components. Self-attention mechanism that allows the model to focus on different parts of the input sequence when generating each output token, capturing dependencies and relationships within the data. Cross-attention mechanism that pays attention to the outputs of the decoder and allows the model to incorporate contextual information from the input sequence into the generation of the output. Feed-forward neural network that applies a position-wise fully connected feed-forward network to each position in the sequence, improving the representation learned from the attention mechanisms. Layer normalization and residual connections that follow each sublayer to stabilize training and improve convergence. Position encoding, which adds positional information to the input embedding so that the model can capture the order of the sequence.

### Experimental settings

While the performance of the segmentation phase of our approach is evaluated on one dataset, the performance of the detection phase is evaluated on four pathological image datasets. The diffusion model serves as a generator for the mask and noise prediction, which is evaluated using the intersection over union (IoU) and Dice coefficient metrics as in the section “Evaluation metrics”. Cancer detection is also evaluated based on the accuracy of the two predicted classes: normal and cancer. The results show competitive or superior performance compared to other methods, supported by both quantitative and qualitative analysis. We investigate different training scenarios. Experiments were performed on a 64-bit Windows 10 Pro system with Python based on PyTorch framework using a CPU E5-2650 v4 @ 2.20 GHz, 128.0 GB RAM and four NVIDIA GTX TITAN X GPUs. The training parameters used for our approach are listed in Table 1.

**Table 1:** Training setting for the breast cancer segmentation network.

Optimizer	Base learning rate	Warmup steps	Training steps	Image size	Batch size	Diffusion steps	Noise schedule
Adamw	1e−4	5000	2e5	128 × 128	16	1000	Linear

The computational resource requirements, in particular the use of four NVIDIA GTX TITAN X GPUs with 12 GB of memory each, reflect the high computational cost of training the Conditional Denoising Diffusion Probabilistic Model (DDPM) and the subsequent feature extraction and fusion steps. While this setup is effective in achieving high segmentation and detection accuracy, it can be challenging in resource-constrained environments, such as clinical settings with limited access to high-end GPUs. To increase the practical relevance of our approach, several strategies can mitigate these requirements. One of the strategies is to reduce the number of diffusion steps (e.g., from 1000 to 200) during inference, as explored in improved DDPM variants^14^. This can significantly reduce the computational overhead with minimal impact on segmentation quality, although it may slightly reduce the precision of the boundaries in complex tissue regions.

### Evaluation metrics

For the segmentation phase, performance is assessed at the pixel level using True Positive (TP), True Negative (TN), False Positive (FP), and False Negative (FN) classifications compared against the ground truth, with class-specific values denoted by a subscript *i* (e.g., $TP_{i}$). These are used to compute the Dice coefficient, $Dice = \frac{2TP_{i}}{2TP_{i} + FP_{i} + FN_{i}}$, and Intersection over Union, $IoU = \frac{TP_{i}}{TP_{i} + FP_{i} + FN_{i}}$, which measure overlap and accuracy of segmented regions. Metrics were calculated over three runs, with the highest result selected for analysis.

In the detection phase, three metrics are employed: accuracy, confidence intervals, and Precision-Recall AUC. Accuracy, defined as $\frac{TP + TN}{TP + TN + FP + FN}$, evaluates overall correctness, while confidence intervals (typically at 95%) assess the reliability of predictions across runs. Precision-Recall AUC, derived from precision ($\frac{TP}{TP + FP}$) and recall ($\frac{TP}{TP + FN}$), quantifies the model’s ability to balance detection quality and completeness across thresholds, especially in imbalanced scenarios. As with segmentation, the highest result from three runs is used for evaluation.

### Datasets

Five data sets are considered, one for the segmentation phase and four for the detection phase.

The BCSS dataset used for the segmentation phase consists of 151 distinct whole slide images (WSIs) stained with hematoxylin and eosin. Each WSI includes outlined regions of interest (ROIs) that encompass representative tissue from the main region classes and textures. In total, 161 ROIs were extracted and annotated by 20 medical students, with ten ROIs labeled by all participants, while the rest were labeled by individual students. The final curated ground truth labels were verified by two experienced pathologists. We used the patches extracted (https://github.com/wizmik12/CRowd_Seg) by Lopez et al.^20^ from the ROIs with a size of $512 \times 512$, which were divided into training (10,173 patches), validation (1,264 patches), and test sets (399 patches). As noted in^20^, the dataset comprises five distinct tissue classes: Tumor, Stroma, Inflammation, Necrosis, and Other. We acknowledge the significant class imbalance inherent in this dataset, where Tumor and Stroma regions are substantially overrepresented compared to the less frequent Necrosis and Other classes. To address this imbalance, we used the strategic patch creation methodology that ensures more equitable representation across all tissue classes in our training, validation, and testing sets.

The BreCaHAD (breast cancer histopathological annotation and diagnosis dataset) dataset^37^ consists of 162 microscopic biopsy images of breast cancer stored in uncompressed (.TIFF) format. Each image is stored in three-channel RGB format with a depth of 8 bits per channel and has a size of $1360 \times 1024$ pixels. This database is only considered for one class, namely the cancer class.

The ICIAR-2018 dataset^36^ includes hematoxylin and eosin (H&E) stained breast histopathology microscopy and whole-slide images. The dataset includes a total of 400 microscopic images evenly divided into four categories: normal, benign, in situ carcinoma, and invasive carcinoma, with 100 images in each class.

The IDC dataset, originally introduced by Cruz-Roa et al.^38^ for the detection of breast cancer, consists of histopathological images available as RGB fields with a size of 50x50 pixels. The original images have not been published, but the dataset contains patches with a zoom factor of 2.5x (4$\upmu \hbox {m}$/pixel). It contains 277,525 patches from 279 subjects, with 28.39% of the patches containing invasive ductal carcinoma cells, while the remainder consists of healthy tissue or non-invasive ductal carcinoma.

The BRACS dataset^39^ comprises 547 WSIs from 189 different patients. In addition, it contains 4,539 regions of interest (ROIs) extracted from 387 WSIs from 151 patients. All slides were scanned with an Aperio AT2 scanner at a resolution of 0.25 $\upmu \hbox {m}$/pixel and a magnification factor of 40$\times$.

## Results and discussion

An analysis of the experimental results, focusing on the evaluation of image segmentation performance across BCSS datasets and four datasets for cancer detection, is presented in this section. Additionally, the influence of various training scenarios on overall performance is discussed, followed by an exploration of the study’s limitations and potential areas for future research.

### Image segmentation performance

The performance of the segmentation phase was evaluated using a publicly available histopathological image dataset, namely BCSS^9^.

**Table 2:** The results of the proposed method in comparison to the state-of-the-art methods for the sub-classes of the BCSS dataset (in percentage).

Methods	Other		Tumor		Stroma		Inflammatory		Necrosis	
	Dice $\uparrow$	IoU $\uparrow$	Dice $\uparrow$	IoU $\uparrow$	Dice $\uparrow$	IoU $\uparrow$	Dice $\uparrow$	IoU $\uparrow$	Dice $\uparrow$	IoU $\uparrow$
DRD-UNet^12^	77.00	62.00	93.00	86.00	91.00	79.00	97.00	84.00	59.00	45.00
ResUNet^47^	73.00	58.00	93.00	85.00	88.00	76.00	91.00	80.00	55.00	41.00
SN-AN (MV)^20^	82.22	–	82.97	–	75.89	–	67.22	–	67.57	–
SN-AN (STAPLE)^20^	82.95	–	81.39	–	75.19	–	65.80	–	54.82	–
SN-AN (CR Image)^20^	84.71	–	83.67	–	75.63	–	77.36	–	75.26	–
Ours	80.75	64.00	83.00	77.50	83.20	76.20	91.20	77.10	67.85	46.20

**Fig. 3:**
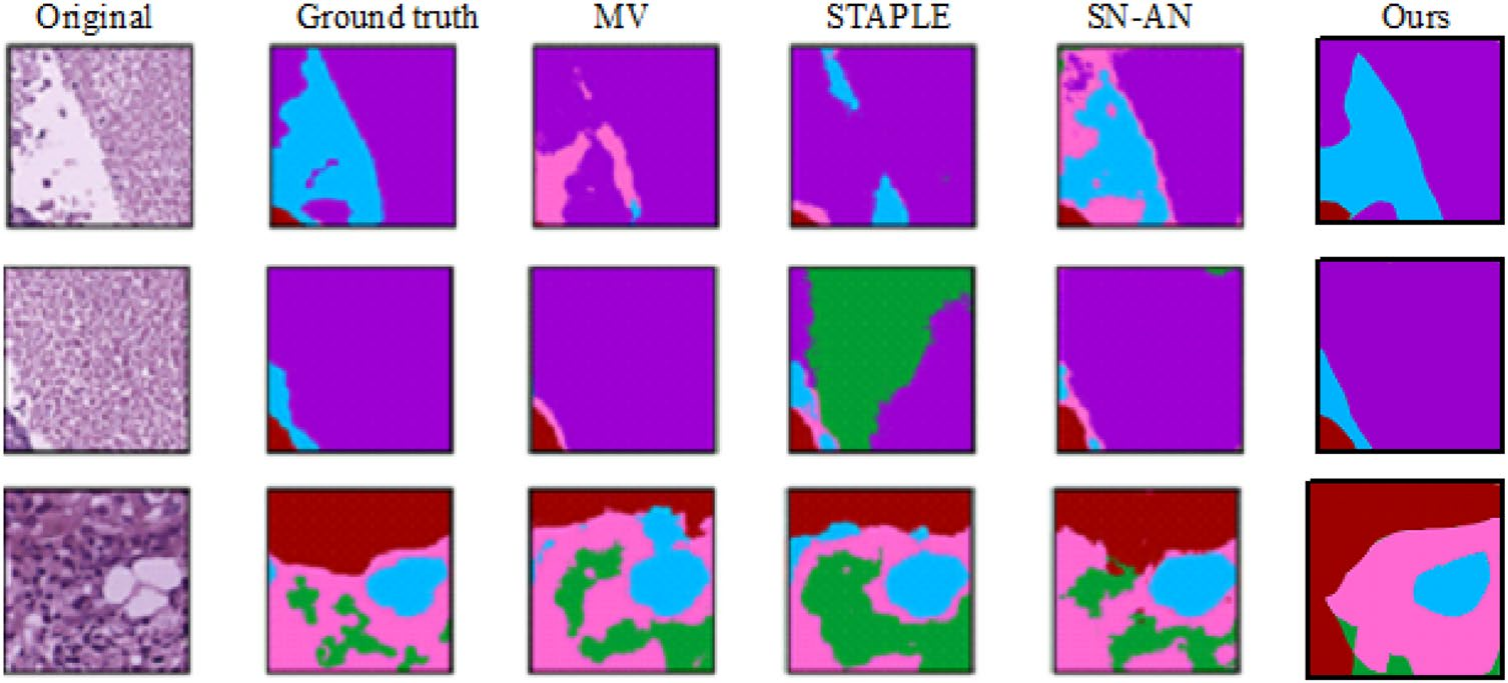
The comparison of segmentation results for images from the BCSS dataset.

The quantitative results for region segmentation on pathological images are summarized in Table 2 that shows the results of the proposed method for subclasses for the BCSS dataset. Although our approach is second in relation to the BCSS dataset, as shown in the table, our approach achieves the best results in two subclasses. The method performs excellently in the segmentation of “Tumor” and “Stroma” regions, which is probably due to their frequency in the dataset and to different morphological features such as dense cellularity or fibrous structures. However, it underperforms in certain subclasses, notably “Necrosis,” where accuracy metrics (e.g., IoU and Dice coefficients) are lower compared to state-of-the-art methods. Two main factors contribute to the lower accuracy in the “Necrosis” subclass. First, the imbalance of classes in the BCSS dataset plays an important role, as mentioned in section “Datasets”. Necrosis regions are underrepresented compared to “Tumor” and “Stroma” and account for a smaller fraction of the annotated patches (e.g.,<5% of the total regions in some samples). This scarcity limits the training data available to the Conditional Denoising Diffusion Probabilistic Model (DDPM) to learn distinctive features of necrotic tissue, resulting in lower generalization and segmentation accuracy. Second, the morphological complexity of necrosis characterized by irregular, amorphous regions with weak boundaries and variable staining poses a challenge for the model. In contrast to “Tumor” regions with clear cellular patterns, necrotic areas often merge with the surrounding tissue, making their precise delineation during the denoising process difficult. The qualitative analysis in Fig. 3 confirms this, as misclassifications in necrosis-heavy samples often occur near indistinct boundaries where the model has difficulty resolving fine-grained noise patterns.

Additional segmentation techniques, such as multi-resolution or multi-scale approaches, could potentially improve accuracy in complex tumor regions. Multi-resolution methods, which process images at varying scales before aggregating outputs, could enhance the DDPM’s handling of fine details (e.g., nuclear morphology in tumors) by capturing both local and global context, as demonstrated in works like DeepLab^48^. Multi-scale architectures, such as those in DETisSeg^11^, excel at resolving hierarchical features, potentially boosting performance in regions with overlapping or subtle boundaries (e.g., tumor-necrosis interfaces). Our current DDPM incorporates a U-Net with attention blocks (section “3.1”), offering some multi-scale benefits.

DDPM was chosen over traditional segmentation methods like U-net^49^ due to its ability to model complex tissue distributions through iterative denoising, capturing subtle patterns in heterogeneous regions like necrosis. Unlike U-net, which relies on deterministic feature extraction, DDPM’s probabilistic approach leverages noise predictions to highlight uncertain boundaries, improving segmentation in necrosis regions with indistinct edges. On the BCSS dataset, DDPM achieved a 3.75% higher Dice coefficient for necrosis (67.85% vs. 64.1% for U-net^49^ ), as its iterative process refines predictions in low-contrast areas, reducing misclassifications.

To validate the impact of attention blocks, we conducted an ablation study on the BCSS dataset, replacing attention blocks with standard U-Net ResNet blocks. The attention-enhanced model achieved a 1.2% higher IoU (77.5% vs. 76.3%) and a 1.1% higher Dice coefficient (83.0% vs. 82.9%) for tumor segmentation, demonstrating that attention mechanisms improve feature focus on complex tissue boundaries, particularly in regions with ambiguous stroma-tumor transitions.

The qualitative results are presented in Fig. 3, illustrating the performance of our method on BCSS datasets. As shown in Fig. 3, our method effectively preserves regions in the multi-class problem and delivers more accurate results compared to the existing methods.

### Cancer detection performance

**Fig. 4:**
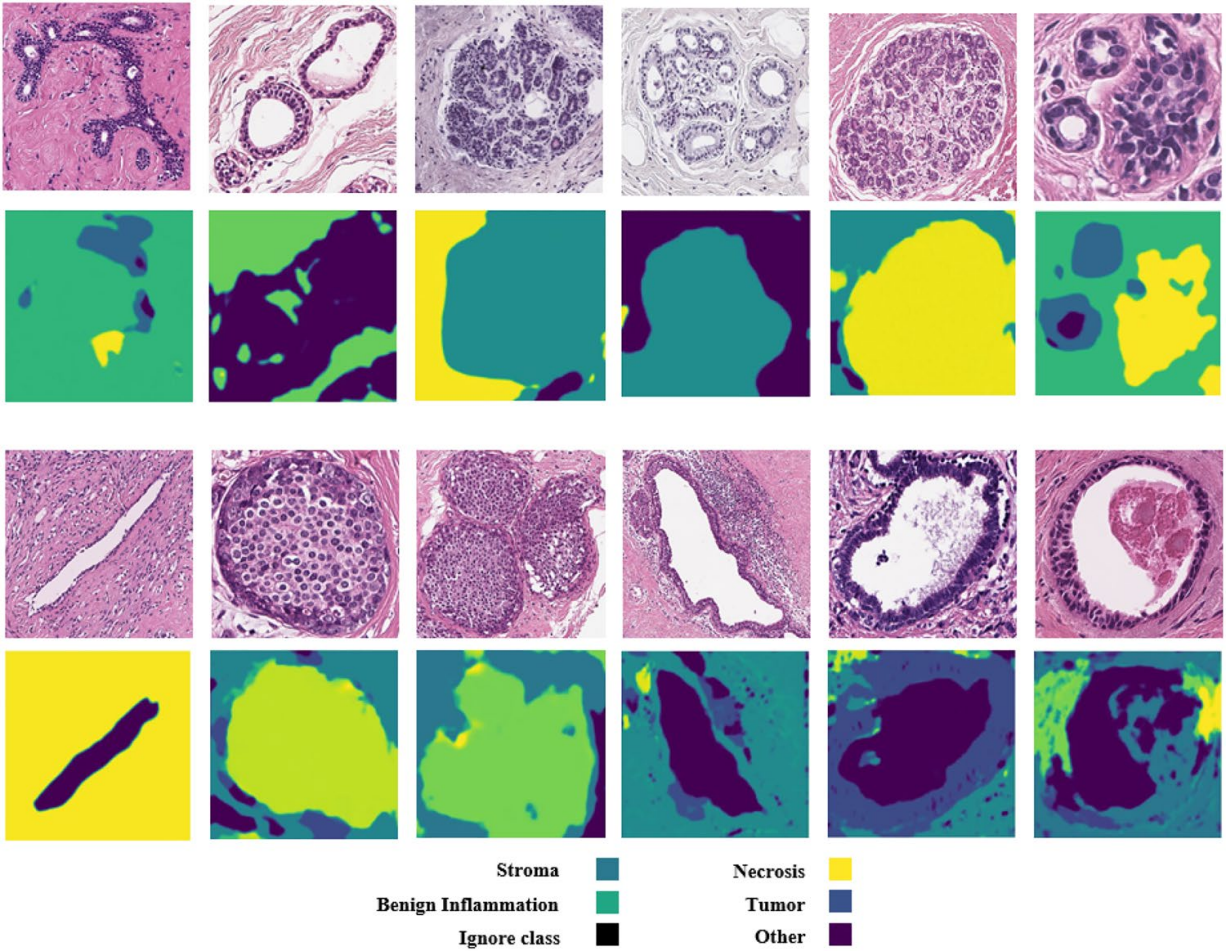
The segmentation outputs for images from the BRACS dataset. The first two rows are normal images and the output of the diffusion models, while the second two rows are cancer images and the output of the diffusion method.

As mentioned earlier, in this study, we proposed a fusion model based on predicted regions, predicted noise, and original images for breast cancer detection. In this section, we present and analyze the obtained results supported by qualitative and quantitative results and analysis of four datasets. Finally, we compare the performance of our approach with several state-of-the-art methods on the datasets used in this study. These datasets, while publicly available, approximate clinical diversity from different hospitals or geographical regions due to variations in imaging protocols (e.g., magnification, staining), patient demographics, and class distributions. The results in terms of BRACS, BreCaHAD, CIAR2018 and IDC datasets are shown in Fig. 4 and Table 3, Fig. 6 and Table 4, Fig. 7 and Table 5, and Fig. 8 and Table 6, respectively. Since some methods are evaluated in the selection of the network and the fusion method, some of these methods are considered in our experiments. To compare the networks, we choose ResNet18^50^, which ranked second in our experiments. For the fusion methods, the simple fusion (concatenation), self-attention^42^ and cross-attention^51^ were selected. “As shown in all tables, the best accuracy is achieved when EfficientNet-B0 and the Transformer Decoder are used as the training network and fusion method, respectively. The transformer decoder fuses features from original images, predicted masks, and noise by using cross-attention to align complementary information (e.g., tumor boundaries from masks with textural details from images) and self-attention to prioritize discriminative features within each modality. This dynamic weighting enhances detection accuracy over static fusion methods. Compared to self-attention (91.61% accuracy) and cross-attention (91.51%), the transformer decoder with three modalities achieves 92.86% accuracy on the BRACS dataset, as its multi-head attention captures long-range dependencies across modalities, reducing errors in complex tumor-stroma interactions.

**Fig. 5:**
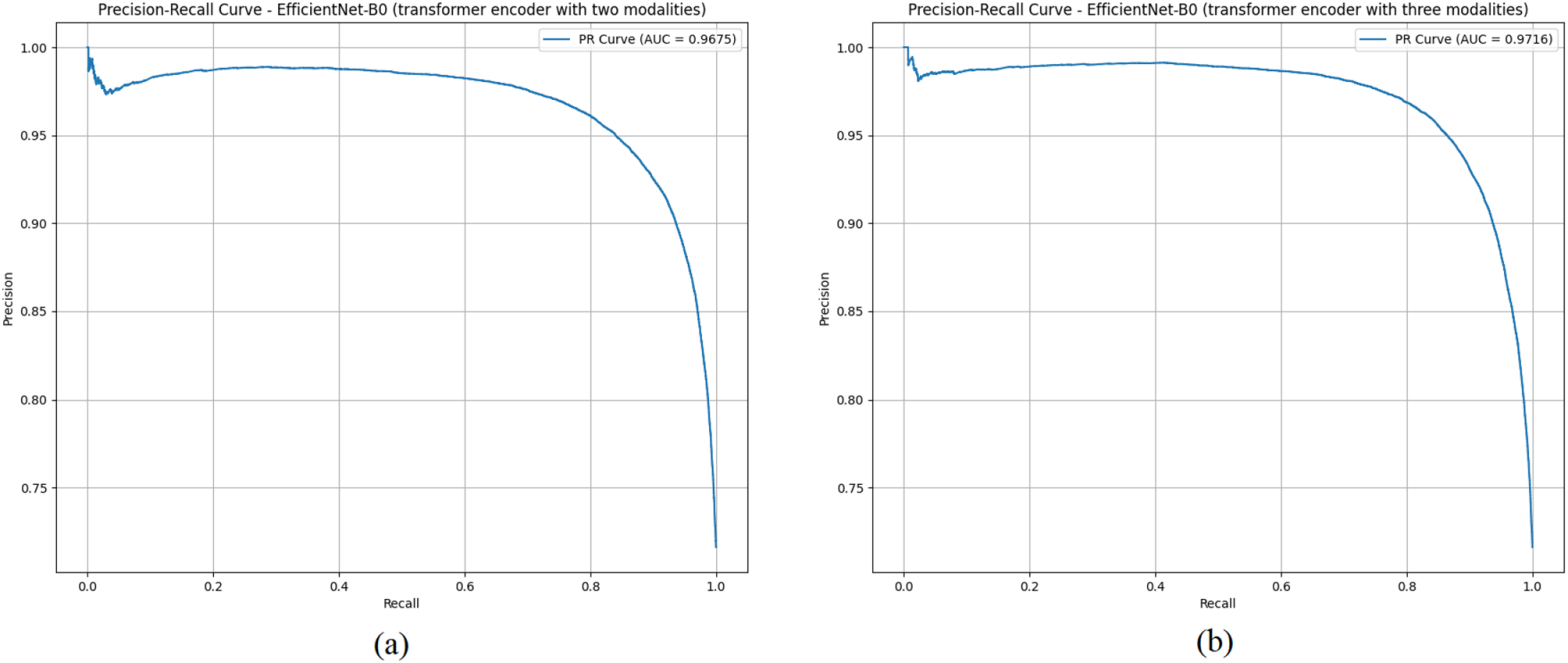
Precision-recall curves comparing the two top-performing methods on the BRACS dataset: (**a**) EfficientNet-B0 (transformer decoder with two modalities) and (**b**) EfficientNet-B0 (transformer decoder with three modalities).

As shown in Table 3, different scenarios are conducted in terms of the BRACS dataset. If our model is based on three modalities with EfficientNet-B0 and transformer decoder, we have an improvement of 2.50% accuracy. Our proposed EfficientNet-B0 with transformer decoder utilizing three modalities achieves the highest performance across all metrics, with an accuracy of 92.86% (CI: 91.98–93.02%) and PR-AUC of 0.971. The table also shows that the predicted region and noise achieved promising results when trained alone. Figure 4 shows the output of the diffusion method for the BRACS dataset in both normal and cancer samples. As can be seen in the figure, the tumor region is present in more cancer samples. Figure 5 illustrates the precision-recall performance of our two highest-performing models. The larger area under the curve for the three-modality model (PR-AUC: 0.971 vs. 0.967) quantitatively confirms this visual observation and aligns with our tabular results, reinforcing the benefit of incorporating the additional modality for improving discriminative power in breast cancer classification from histopathological images.

**Table 3:** The results of the proposed method compared with different scenarios in terms of the BRACS dataset (region of interest (ROI)).

Methods	Accuracy (%)	95% CI	PR-AUC
Wang et al.^52^	82.08	–	–
Resnet (original image)	90.36	[88.92–91.80]	0.912
Resnet (diffusion output)	86.28	[84.61–87.95]	0.875
Resnet (diffusion noise)	85.60	[83.90–87.30]	0.869
Resnet (simple fusion with three modalities)	91.51	[90.15–91.87]	0.937
Resnet (self-attention-Based Fusion Model)	91.61	[90.26–91.96]	0.951
Resnet (Cross-Attention Fusion Model)	91.51	[90.15–91.87]	0.948
Resnet (transformer decoder-based fusion)	91.84	[90.51–91.17]	0.954
EfficientNet-B0 (original image)	90.82	[89.41–91.23]	0.938
EfficientNet-B0 (cross attention)	92.40	[91.11–92.69]	0.970
EfficientNet-B0 (transformer decoder with two modalities)	92.06	[90.74–92.38]	0.967
EfficientNet-B0 (transformer decoder with three modalities)	**92.86**	**[91.98–93.02]**	**0.971**

**Fig. 6:**
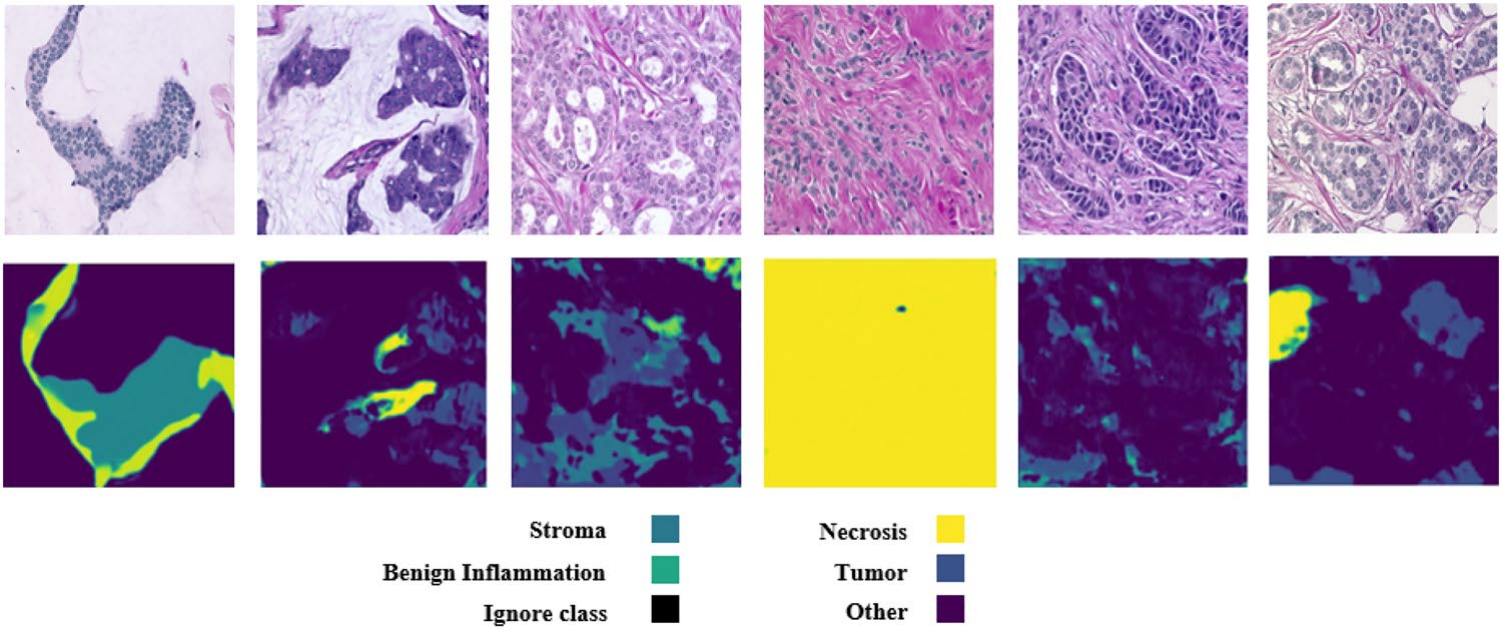
The segmentation outputs for images from the BreCaHAD dataset. The dataset includes only cancer samples.

As can be seen in Table 4, our approach correctly recognizes all samples with respect to the BreCaHAD dataset. It should be noted that the dataset contains only cancer samples. Also, we used our trained model on the BRACS dataset to detect the entire BreCaHAD dataset. Figure 6 shows the result of the diffusion method with respect to the dataset. As can be seen in the figure, the tumor is detected in most of the samples.

**Table 4:** The results of the proposed method compared with the state of the art of the BreCaHAD dataset (%).

Method	Accuracy
Dilated Residual (DR) model^53^	88.70
EfficientNet-B0 (Original image)	98.77
EfficientNet-B0 (cross attention)	99.39
EfficientNet-B0 (transformer decoder-based fusion)	**100**

**Figure 7:**
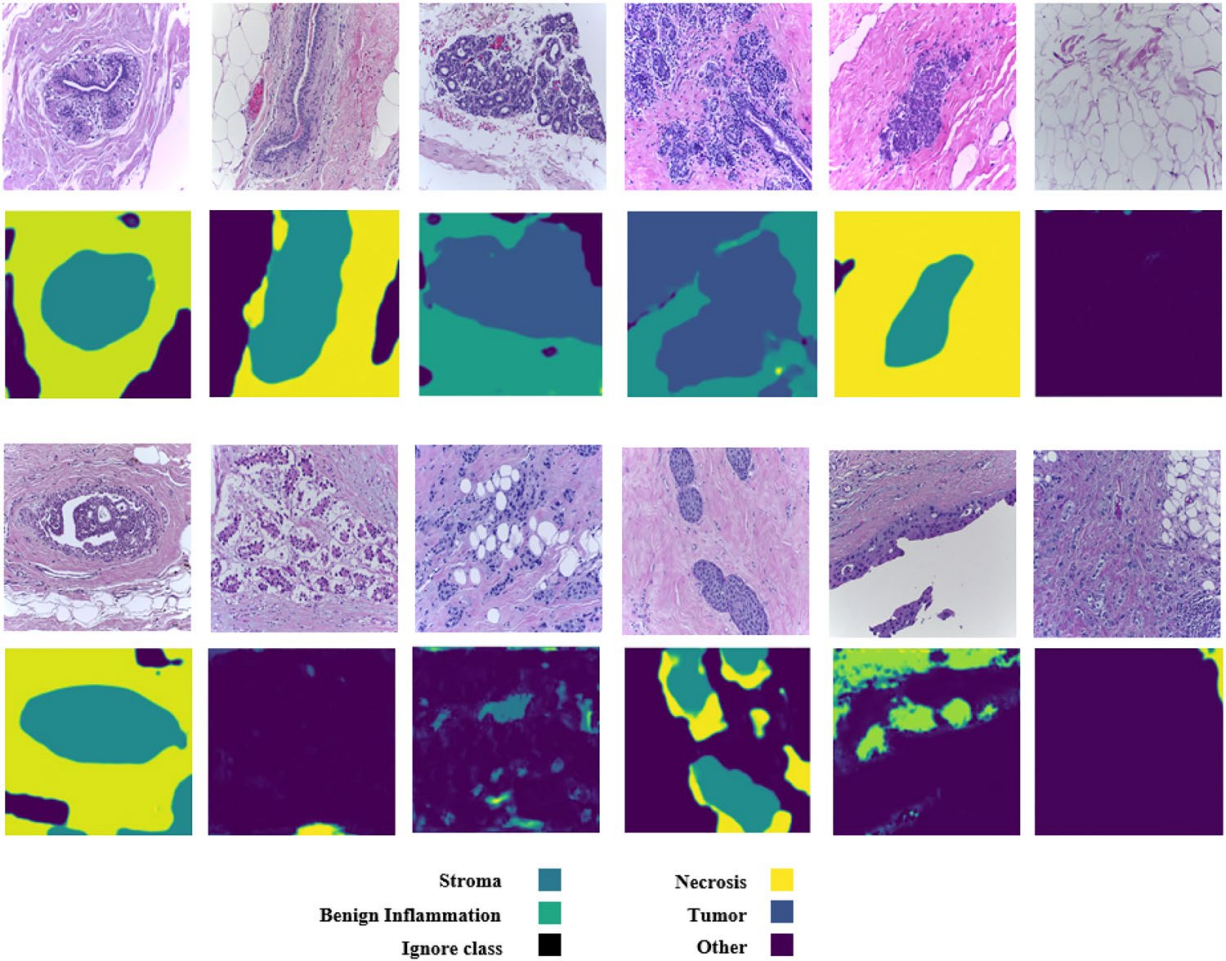
The segmentation outputs for images from the ICIAR2018 dataset. The first two rows are normal images and the output of the diffusion models, while the second two rows are cancer images and the output of the diffusion method.

In relation to the ICIAR2018 dataset, two scenarios are performed as shown in Table 5. A model trained with the BRACS dataset and a model trained with the ICIAR2018 dataset. As can be seen from the table, the second scenario achieved better results. Compared to the state of the art, our approach ranks second with a 0.29% difference to the first one. Similar to the BRACS dataset, the diffusion method effectively identifies tumor regions in the ICIAR2018 dataset, as shown in Fig. 7.

**Table 5:** The results of the proposed method compared with the state of the art of the ICIAR2018 dataset (%).

Method	Trained by BRACS and evaluated with ICIAR2018	Train and test with the same dataset
	Accuracy	Accuracy
Kassani et al.^54^	–	95.00
Vizcarra et al.^55^	–	92.00
Bhowal et al.^29^	–	96.00
Majumdar et al.^27^	–	96.95
EfficientNet-B0 (Original image)	66.67	93.33
EfficientNet-B0 (cross attention)	71.67	95.00
EfficientNet-B0 (transformer decoder with three modalities)	**73.33**	**96.66**

**Fig. 8:**
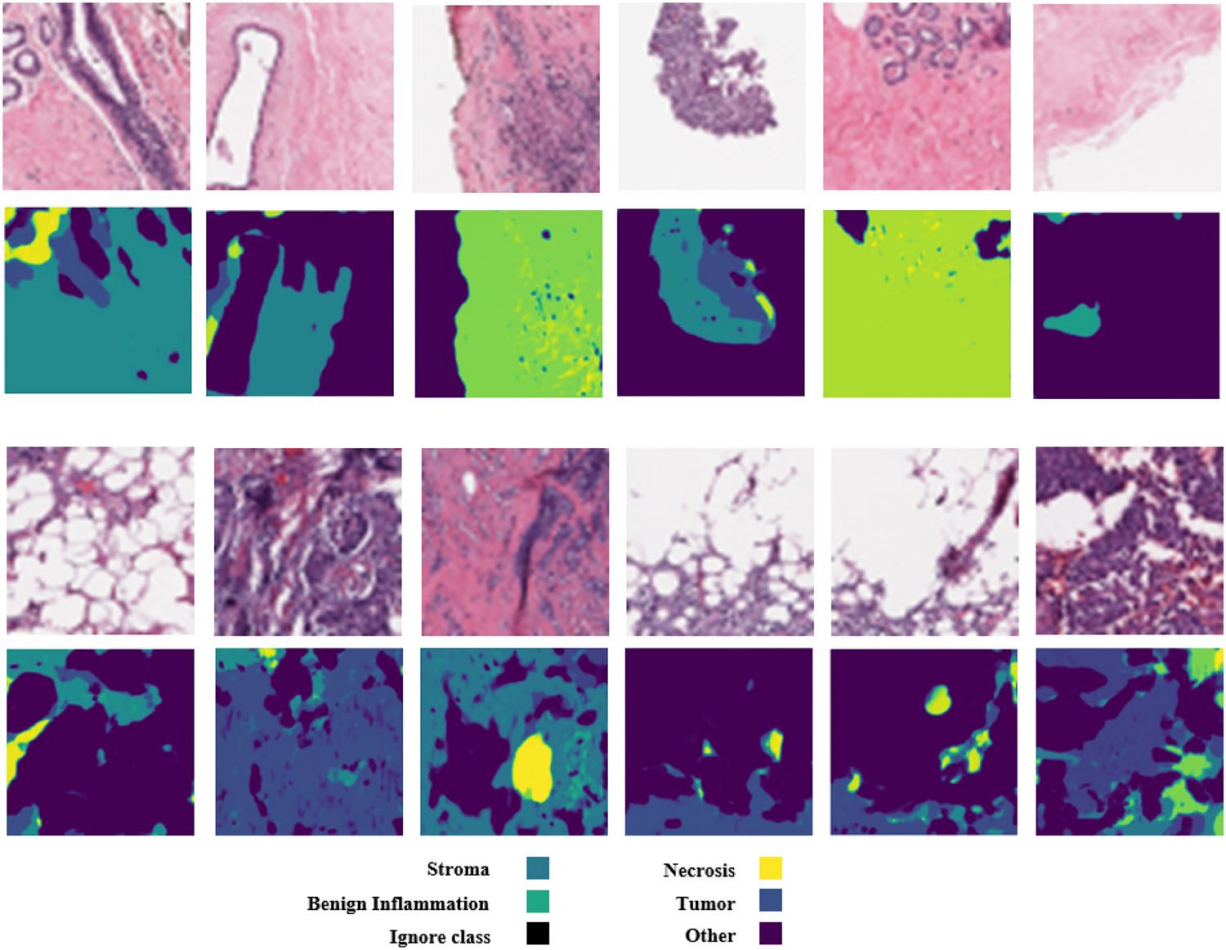
The segmentation outputs for images from the IDC dataset. The first two rows are normal images and the output of the diffusion models, while the second two rows are cancer images and the output of the diffusion method.

Table 6 shows the result of our proposed approach compared to the state of the art in terms of the IDC dataset. Although our approach achieves the second rank, our scenarios for training and evaluation differ from the first rank. We used a cross-validation scenario to have all the data for both training and testing, while the first rank randomly splits the data into training and testing data. Also, Fig. 8 shows the results of the diffusion method on the dataset.

**Table 6:** The results of the proposed method compared with the state of the art of the IDC dataset (%).

Method	Accuracy
Romero et al.^56^	89.00
Singh and Kumar^57^	80.17
Humayun et al.^58^	**91.00**
EfficientNet-B0 (Original image)	88.41
EfficientNet-B0 (cross attention)	88.77
EfficientNet-B0 (transformer decoder-based fusion)	89.59

### Training scenarios

In the first scenario, each modality (original image, predicted region mask, and predicted noise) is trained separately, and the results with respect to the BRACS dataset are shown in Table 3. Training the original image along with EfficientNet-B0 yielded an accuracy of 90.82%, while the diffusion output (predicted mask) and noise achieved 86.28% and 85.60%, respectively. These results suggest that the original image carries the most discriminative information, likely due to its rich spatial and textural details, whereas the predicted mask and noise provide complementary but less comprehensive features when used independently. Fusing all three modalities with the transformer decoder improved accuracy to 92.86%, a 2.04% gain over the original image alone, demonstrating the value of integrating multi-modal data. A two-modality experiment combining the original image and predicted mask achieved 92.06%, indicating that the mask adds significant structural context, while the noise’s marginal contribution (e.g., 92.86% vs. 92.06%) reflects its role in refining subtle features rather than driving primary detection. The performance difference here highlights that generalizability improves with fusion, as the model leverages diverse representations to mitigate overfitting to a single modality, though the computational cost increases. The fusion of three modalities (original image, predicted mask, and noise) increases computational complexity compared to single-modality approaches. On our setup (section “Experimental settings”), processing a single image with EfficientNet-B0 and transformer decoder takes 0.6 s for one modality, 1.1 s for two modalities, and 1.3 s for three modalities during inference. While the three-modality fusion achieves the highest accuracy (92.86%, Table 3), the 0.7-s increase over single-modality processing may challenge real-time clinical applications.

In the second scenario, we evaluated the ICIAR2018 dataset when our model was trained with the BRACS dataset, as shown in Table 5, which shows promising results. The model was trained on the BRACS dataset and evaluated on ICIAR-2018, yielding 73.33% accuracy (Table 5, second column), a 23.33% decrease compared to when train and test are based on the same dataset. This gap highlights generalizability challenges: BRACS, with its diverse subtype representation (section “Datasets”), trains a robust model for cancer detection, but its mismatch with ICIAR-2018’s class balance and imaging characteristics (e.g., ROI size) reduces transferability. Ablation studies reinforce this trend, with BRACS-trained variants like cross-attention (71.67%) and original-image-only (66.67%) also underperforming. Although cross-dataset performance decreases due to domain shift stemming from differences in staining, magnification, and ROI selection our model maintains promising accuracy and robustness (see Table 5. This highlights the benefit of tumour-guided model selection in compensating for such differences, even without direct domain adaptation.

### Limitations

As illustrated in Fig. 3, our method exhibits lower accuracy when processing multi-class samples, particularly when the number of classes is small, resulting in segmentation errors. Additionally, while diffusion models are known for their capability to utilize different realizations to enhance final results, our approach utilized only a single realization, excluding other potential realizations from our results. Incorporating multiple realizations could potentially improve outcomes, and this aspect warrants further investigation. Practical deployment of our method in clinical settings faces challenges due to the high computational cost of DDPM, requiring four NVIDIA GTX TITAN X GPUs for training (section “Experimental settings”). Inference with 1000 diffusion steps takes approximately 2.5 s per image, which may be prohibitive for real-time pathology systems with limited hardware. Additionally, the model’s reliance on large, annotated datasets like BCSS may limit applicability in resource-constrained environments with smaller or unannotated datasets. To enhance real-world applicability, reducing diffusion steps to 200, as suggested by Nichol et al.^14^, could lower inference time to <0.5 s with a minimal 2–3% IoU drop, and model pruning could reduce memory demands by up to 50%, making deployment feasible on mid-range GPUs. Future work will validate these optimizations across datasets like BCSS and BRACS for a more efficient model.

## Discussion and conclusions

This study demonstrates that integrating DDPM-based segmentation with multi-modal fusion via EfficientNet-B0 and a transformer decoder significantly enhances breast cancer detection. The method’s robustness across four datasets highlights its potential for computational pathology. However, computational costs and generalizability challenges remain. Actionable next steps include optimizing DDPM with fewer diffusion steps, implementing ensemble diffusion sampling, and fine-tuning on diverse cohorts to improve robustness. For clinical integration, the model can be deployed as a decision-support tool in pathology workflows, providing real-time tumor region highlighting to assist pathologists, particularly in resource-limited settings, by integrating with existing digital slide scanners after pruning for mid-range hardware compatibility.

This work not only represents a promising tool for computational pathology but also illustrates the potential of integrating different data representations to achieve more accurate and reliable diagnostic results. The approach has significant implications for clinical applications. The combination of DDPM-based segmentation and multimodal fusion improves the model’s ability to deal with inter-patient variability, a critical factor in breast cancer histopathology, where tissue morphology, staining intensity, and tumor microenvironment vary greatly from patient to patient. The success of the model on various datasets such as BRACS (547 WSIs, multiple subtypes) and ICIAR2018 (four categories) suggests that it is robust to variations in cancer subtypes and histologic patterns, as the fusion of original images, masks, and noise captures both global and local features that withstand patient-specific differences. However, differences in performance (e.g., 94.50% vs. 97.50% for ICIAR2018, Table 5) when applying a BRACS-trained model to unseen data highlight the challenges of fitting. Unknown datasets with novel staining protocols or rare subtypes (e.g., triple negative cases underrepresented in training) could compromise accuracy if not accounted for. To mitigate this, strategies such as transfer learning, fine-tuning the EfficientNet-B0 and Transformer decoder on small, patient-specific datasets, or domain adaptation techniques could adapt the model to new data distributions, improving generalizability without the need for extensive retraining. In the clinical setting, this adaptability could reduce false negative results in different populations and thus increase diagnostic reliability, although this requires access to annotated samples or computational resources for fine-tuning, which may be limited in some cases.

Future work will address identified limitations through targeted optimizations. To mitigate computational constraints, we will explore distilled DDPM variants, reducing diffusion steps from 1000 to 200, which could cut inference time by 80% with minimal performance loss^14^. To leverage multiple realizations in diffusion, we will implement ensemble diffusion sampling, averaging predictions from multiple noise realizations to improve segmentation accuracy, as suggested by Amit et al.^15^. To reduce segmentation inaccuracies, particularly in multi-class scenarios (section “Limitations”), we will incorporate class-balanced loss functions and synthetic data augmentation. We plan to improve generalizability by validating the model on larger, multi-center cohorts from diverse populations. The framework’s applicability will be tested on other cancer types, such as lung or prostate cancer. We will investigate real-time integration into pathology systems by collaborating with clinicians to develop decision-support tools.

### Ethical considerations

The datasets employed in this study, BCSS, BreCaHAD, ICIAR-2018, IDC, and BRACS, are publicly available resources that were specifically curated for research purposes. Each of these datasets has undergone the necessary anonymization procedures and received appropriate ethical clearances prior to their public release. We strictly adhered to the terms of use for each dataset and maintained the anonymized nature of the data throughout our research process. As these are established research datasets, detailed information regarding patient data anonymization processes, informed consent procedures, and compliance with regulations such as HIPAA or GDPR is documented in the original publications referenced. No additional patient data was collected for this study, and no attempt was made to re-identify any samples. Our data handling procedures focused on secure storage of the publicly available datasets, with access limited to authorized research team members only. For specific details regarding the anonymization protocols and regulatory compliance for each dataset, readers are directed to the original publications where these aspects are thoroughly documented.

## Data Availability

The datasets analysed during the current study are available in the BCSS dataset: https://github.com/wizmik12/CRowd_Seg, BreCaHAD: https://figshare.com/articles/dataset/BreCaHAD_A_Dataset_for_Breast_Cancer_Histopathological_Annotation_and_Diagnosis/7379186, ICIAR-2018: https://iciar2018-challenge.grand-challenge.org/Dataset/, IDC dataset: https://www.kaggle.com/datasets/kasikrit/idc-dataset, and BRACS dataset: https://www.bracs.icar.cnr.it/. Also, all data analysed (mentioned links) during this study are included in the published article.
